# Matrix metalloproteinase MMP-8, TIMP-1 and MMP-8/TIMP-1 ratio in plasma in methicillin-sensitive *Staphylococcus aureus* bacteremia

**DOI:** 10.1371/journal.pone.0252046

**Published:** 2021-05-27

**Authors:** Erik Forsblom, Taina Tervahartiala, Eeva Ruotsalainen, Asko Järvinen, Timo Sorsa

**Affiliations:** 1 Division of Infectious Diseases, Inflammation Center, University of Helsinki and Helsinki University Hospital, Helsinki, Finland; 2 Division of Infectious Diseases, Inflammation Center, Helsinki University Central Hospital, Meilahti Triangle Hospital, Helsinki, Finland; 3 Department of Oral and Maxillofacial Diseases, University of Helsinki and Helsinki University Hospital, Helsinki, Finland; 4 Division of Periodontology, Department of Dental Medicine, Karolinska Institutet, Huddinge, Sweden; Indiana University School of Medicine, UNITED STATES

## Abstract

**Background:**

Matrix metalloproteinase-8 (MMP-8) and tissue inhibitor of metalloproteinases-1 (TIMP-1) have been shown to predict prognosis in sepsis. However, MMP-8 and TIMP-1 in *Staphylococcus aureus* bacteremia (SAB) lacks evaluation and their role in the pathogenesis of SAB is unclear.

**Methods:**

MMP-8 and TIMP-1 and MMP-8/TIMP-1 molar ratio were determined at days 3, 5 and 28 from positive blood cultures in patients with methicillin-sensitive SAB and the connection to disease severity and early mortality was determined.

**Results:**

Altogether 395 SAB patients were included. Patients with severe sepsis or infection focus presented higher MMP-8 levels at day 3 and 5 (p<0.01). Higher day 3 and 5 MMP-8 levels were associated to mortality at day 14 and 28 (p<0.01) and day 90 (p<0.05). Day 3 MMP-8 cut-off value of 203 ng/ml predicted death within 14 days with an area under the curve (AUC) of 0.70 (95% CI 0.57–0.82) (p<0.01). Day 5 MMP-8 cut-off value of 239 ng/ml predicted death within 14 days with an AUC of 0.76 (95% CI 0.65–0.87) (p<0.001). The results for MMP-8/TIMP-1 resembled that of MMP-8. TIMP-1 had no prognostic impact. In Cox regression analysis day 3 or 5 MMP-8 or day 3 MMP-8/TIMP-1 had no prognostic impact whereas day 5 MMP-8/TIMP-1 predicted mortality within 14 days (HR, 4.71; CI, 95% 1.67–13.3; p<0.01).

**Conclusion:**

MMP-8 and MMP-8/TIMP-1 ratio were high 3–5 days after MS-SAB diagnosis in patients with an infection focus, severe sepsis or mortality within 14 days suggesting that matrix metalloproteinase activation might play a role in severe SAB.

## Introduction

*Staphylococcus aureus* bacteremia (SAB) causes considerable morbidity and mortality which still ranges from 9%– 34% [1–3]. Prognosis of SAB is worsened by high age and comorbidity [2], severe sepsis [3], endocarditis [4] and methicillin-resistance [5]. However, prognosis has improved by better infection management through infectious specialist consultation (ISC) [6,7].

*Staphylococcus aureus* express surface structures of which cell-wall peptidoglycan is among the most important [8]. *Staphylococcus aureus* peptidoglycan induce matrix metalloproteinases (MMPs) levels in rat tissue [9] and induce elevated concentrations of neutrophil originated MMPs in human blood [10]. Furthermore, *Staphylococcus aureus* peptidoglycan displays inflammatory properties in human blood such as release of cytokines and the upregulation of tissue factor in human monocytes [11,12]. MMPs and their tissue inhibitors (TIMPs) have been studied as promising new diagnostic and prognostic biomarkers for critically ill and septic patients [13–15]. Structurally related but genetically distinct MMPs are zinc dependent proteolytic enzymes that control intra- and extracellular matrix proteins [16,17] and enhance migration of immune cells and pro-inflammatory and cytokine responses [18]. MMPs degrade almost all extracellular and basement membrane proteins as well as non-matrix bioactive molecules and mediators including α1-antitrypsin, bradykinin, insulin-receptor and complement component and thereby regulate immune responses [18,19]. The activities of MMPs are regulated by TIMPs which is the most potent endogenous inhibitor of MMP-8 and their ratio has been used in evaluation of effective pharmacological treatments [20]. In severe inflammation and critical illness the balance of MMPs and inhibitory TIMPs may become disturbed [21]. Among the most important MMP subgroups are the collagenases (MMP-1, -8, -13) and the gelatinases (MMP-2, -9) [22]. This data suggests that MMP activation could have a role in many pathogenic processes of complicated SAB and might be one potential therapeutic target.

Previous reports have demonstrated an upregulation of circulating levels of MMPs and TIMPs in critically ill or septic patients compared to controls. Furthermore, high levels of MMP-8, MMP-9 and TIMP-1 have been connected to mortality in septic or septic shock patients [13,23–34]. Although MMPs and TIMPs have been extensively evaluated in sepsis, only a few studies report inclusion of bacteremia patients [25–27,31] and none of them show data separately from them.

The objective of the present study was to evaluate plasma levels of MMP-8, TIMP-1 and MMP-8/TIMP-1 ratio in methicillin-sensitive SAB (MS-SAB) and their possible role in disease pathogenesis by evaluating their predictive value on disease severity and mortality. All patients were provided with formal bedside ISC which guaranteed proper clinical management including non-delayed proper antibiotic therapy onset from the first day of SAB.

## Materials and methods

### Ethics statement

The institutional review board of Helsinki University Hospital, The Ethical committee of Helsinki University Hospital and each study site (Helsinki University Hospital, Helsinki; Kuopio University Hospital, Kuopio; Oulu University Hospital, Oulu; Tampere University Hospital, Tampere; Turku University Hospital, Turku; Satakunta Central Hospital, Pori; North Karelian Central Hospital, Joensuu; Lapland Central Hospital, Rovaniemi; Peijas Hospital, Vantaa; Maria Hospital, Helsinki; Malmi Hospital, Helsinki; Jorvi Hopsital, Espoo) approved the study (study number 82/99) and a written informed consent was provided from each patient [35].

### Settings and study population

This was a prospective multicenter study. Adult patients with blood cultures positive for *S*. *aureus* were included from seven central hospitals and five university hospitals in Finland from January 1999 to May 1999 and January 2000 to August 2002. A total of 430 SAB patients were included. Median time-interval between blood culture sampling and study inclusion was three days. All patients were provided with formal bedside ISC. The following were exclusion criteria: age < 18 years, pregnancy, breastfeeding, imprisonment, epilepsy, bacteremia 28 days prior to the study, poly-microbial bacteremia and meningitis [35]. No cases of methicillin-resistant *S*. *aureus* (MRSA) were included. Data collection included gender, age, bacteremia acquisition, time to defervescence (axillary temperature below 37.5°C), underlying diseases and length and administration route of any antibiotic therapy. Parameters required for Pitt bacteremia score calculation i.e. mental status, vital signs, any mechanical ventilation and recent cardiac arrest [36,37] as well as any ICU treatment were recorded. Infection focus documentation was based on clinical suspicion or verified by radiological, bacteriological, or pathological investigations. Plasma samples for MMP-8 and TIMP-1 concentrations were drawn at days 3, 5 and 28 from the positive blood culture sampling. Primary endpoint was mortality at 14 days. Secondary endpoints were mortality at 28 and 90 days as well as disease severity, prevalence of deep infection foci and time to defervescence.

### Follow-up time period

Patients were followed prospectively for at least 90 days. Patients transferred to other hospitals were followed from patient records and direct contact to the hospital. Patients who were not hospitalized at 28 days had an additional laboratory appointment arranged to enable laboratory sampling at 28 days as well as an outpatient clinic follow-up visit at 90 days.

### Definitions

Bacteremia with the first positive blood culture for *S*. *aureus* obtained 48 h after hospital admission was defined as healthcare-associated (HA) SAB. The same applied when the patient had undergone hemodialysis within the preceding two months or remained in a long-term care facility. McCabe’s criteria were applied for classification of underlying diseases [38]. Severe sepsis was classified as sepsis in combination with hypotension, hypo-perfusion, or organ failure [39]. Endocarditis was defined according to the modified Duke criteria [40]. Deep infection foci included mediastinitis, pneumonia, endocarditis, purulent arthritis, osteomyelitis, deep-seated abscess, and any foreign-body infection. Deep infection foci were documented either based on clinical suspicion or verified by bacteriological, radiological or pathological findings [41].

### Antibiotic therapy

Anti-staphylococcal penicillin (cloxacillin) was the first-line antibiotic therapy, whereas patients with contraindications for penicillin were provided with especially cefuroxime but also ceftriaxone, clindamycin or vancomycin. Fluoroquinolone (levofloxacin) and/or rifampicin were provided as additional therapy [35]. The dosages for intravenously administered antibiotics were as follows; cloxacillin 2 g q 4 h, cefuroxime 1.5 g q 6 h, ceftriaxone 2 g q 24 h clindamycin 600 mg q 6–8 h or vancomycin 1 g q 12 h. For oral treatment the dosages were as follows; levofloxacin 500 mg q 24 h for patients under 60 kg and 500 mg q 12 h for patients over 60 kg in weight and/or for rifampicin 450 mg q 24 h for patients under 50 kg and 600 mg q 24 h for patients over 50 kg in weight. Patients with diagnosed deep infection foci had intravenous antibiotic therapy for at least 28 days, whereas in the absence of any deep infection, 14 days was regarded as a proper length. For patients with renal or hepatic dysfunction the antibiotic doses were adjusted as recommended by the manufacturers.

### MMP-8, TIMP-1 and C-reactive protein analysis

Plasma samples for MMP-8, TIMP-1 and CRP measurements were taken on days 3, 5 and 28 after positive blood cultures. Samples for MMP-8 and TIMP-1 analyzes were centrifuged at 2000 *g* for 10 min and the supernatants were stored at −80°C until analysis. We measured MMP-8 plasma concentration by time-resolved immuno-fluorometric assay (IFMA) [31,42] by using monoclonal MMP-8 specific antibodies 8708 and 8706 (Medix Biochemica, Kauniainen, Finland) for catching and tracing. Europium chelate was applied for labelling the tracer antibody. TIMP-1 analysis was done by using commercially available enzyme-linked immunosorbent assay according to the manufacturer’s instructions (Biotrak ELISA System; Amersham Biosciences, Buckinghamshire, UK). Concentrations are reported as ng/ml. Detection limits for MMP-8 and TIMP-1 are 0.08 ng/ml and 1.25 ng/ml, respectively. The levels of MMP-8 and TIMP-1 were denoted as ng/ml and for calculation of MMP-8/TIMP-1 molar ratio the levels were converted to mol/l [43,44]. The MMP-8 and TIMP-1 were properly stored (−80°C storage) after collection and analyzed in 2015–2016. Laboratory quality testing ensured that MMP and TIMP remained stable at –80°C and hence the standard of the laboratory samples were guaranteed despite longtime storage. Plasma C-reactive protein was subjected to automatic immune-turbidometric using analyzers 917 or Modular PP-analyzer (Hitachi Ltd, Tokyo, Japan) and Tina-quant CRP reagents (Roche Diagnostics, Tina-quant). The normal value of CRP concentration was < 10 mg/L for both methods. The anti-coagulant used for the blood sampling of MMP-8, TIMP-1 and CRP was heparinate or EDTA.

### Statistical analysis

Data are presented either as absolute values and percentages or as mean with standard deviation. Categorical variables are compared with *Pearson’s X* ^*2*^ -test whereas non-categorical variables are analyzed with *Student’s t-test*. Odds ratios (OR) with 95% confidence intervals (CI) were calculated. The discriminative power of MMP-8, TIMP-1, MMP-8/TIMP-1 and CRP in predicting mortality was evaluated by receiver operating characteristic (ROC) curves. The area under the curve (AUC) was calculated. Youden index was defined as the sensitivity and specificity sum with the highest value or the ROC-curve point equally maximizing both sensitivity and specificity values to locate the cut-off point. The ROC-curve derived cut-off point results were applied for Cox regression model (proportional regression) predicting mortality. Generalized linear model (GLM) repeated measure analyze was applied for comparison of MMP-8, TIMP-1 and MMP-8/TIMP-1 throughout the 90 days follow-up period for patients who deceased versus those that survived. Univariate factors with p <0.1 were allowed for the Cox proportional regression model. All tests were two-tailed and p <0.05 was considered as significant. Analyzes were done using SPSS 12.0 (SPSS Inc., Chicago, IL, USA).

## Results

### Patient characteristics

Altogether 430 SAB patients were included. All methicillin-resistant cases were excluded (N = 6). Due to missing plasma samples the results for 395 (day 3), 378 (day 5) and 315 (day 28) patients are presented. However, no patients were lost during the follow-up time-period for other reasons that death. The mean MMP-8 and TIMP-1 levels at day 3, 5 and 28 from positive blood culture were stratified according to patient characteristics (Table 1). Patient demographics and underlying conditions had no impact on MMP-8 or TIMP-1 levels on day 3 or 5 with the exception of chronic renal failure that was associated to lower MMP-8 (p<0.05) and higher TIMP-1 (p<0.01) values. Patient demographics and underlying conditions did not affect MMP-8 levels on day 28 but significantly higher TIMP-1 levels were associated to male sex (p<0.05), age > 60 years (p<0.01) and ultimately or rapidly fatal underlying disease (p<0.001) as well as coronary artery disease (p<0.01), chronic renal failure (p<0.001), diabetes mellitus (p<0.001) and alcoholism (p<0.05) (Table 1).

**Table 1:** Plasma matrix metalloproteinase-8 (MMP-8) and tissue inhibitor of metalloproteinase-1 (TIMP-1) concentrations (ng/ml) at day 3 (N = 395), day 5 (N = 378) and day 28 (N = 315) from positive blood cultures in patients with methicillin-sensitive *Staphylococcus aureus* bacteremia stratified according to patient characteristics, severity of illness and outcome.

Patient parameters	N (%)	Day 3						Day 5						Day 28					
		MMP-8 (ng/ml)			TIMP-1 (ng/ml)			MMP-8 (ng/ml)			TIMP-1 (ng/ml)			MMP-8 (ng/ml)			TIMP-1 (ng/ml)		
		Parameter present ^1^	Parameter absent ^1^	p- value	Parameter present ^1^	Parameter absent ^1^	p- value	Parameter present ^1^	Parameter absent ^1^	p- value	Parameter present ^1^	Parameter absent ^1^	p- value	Parameter present ^1^	Parameter absent ^1^	p- value	Parameter present ^1^	Parameter absent ^1^	p- value
**Demographics**																			
Male sex	248 (63)	221 ±192	242 ±238	NS	92 ±14	89 ±21	NS	218 ±192	222 ±232	NS	90 ±16	91 ±20	NS	111 ±128	117 ±152	NS	77 ±18	73 ±19	*
Age > 60 years	201 (51)	249 ±228	208 ±188	NS	91 ±17	91 ±17	NS	236 ±214	202 ±201	NS	91 ±18	90 ±17	NS	132 ±149	119 ±124	NS	81 ±18	71 ±18	**
Healthcare-associated	210 (53)	204 ±181	257 ±236	NS	92 ±18	90 ±16	NS	194 ±178	250 ±237	NS	91 ±18	90 ±17	NS	115 ±135	111 ±139	NS	74 ±17	77 ±19	*
**Underlying condition**																			
Healthy-nonfatal ^**A**^	284 (72)	234 ±211	215 ±208	NS	90 ±17	93 ±16	NS	223 ±212	210 ±199	NS	89 ±18	94 ±16	NS	109 ±128	129 ±164	NS	72 ±18	86 ±18	***
Ultimately-rapidly ^**A**^	112 (28)	215 ±208	234 ±211	NS	93 ±16	90 ±17	NS	210 ±199	223 ±212	NS	94 ±16	89 ±18	NS	129 ±164	109 ±128	NS	86 ±18	72 ±18	***
Coronary artery disease	103 (26)	217 ±200	233 ±214	NS	93 ±13	90 ±18	NS	219 ±194	219 ±213	NS	93 ±15	90 ±18	NS	115 ±127	113 ±140	NS	82 ±16	73 ±19	**
Chronic renal failure ^**B**^	57 (14)	174 ±169	238 ±215	*	98 ±21	90 ±16	**	166 ±155	228 ±215	NS	95 ±19	90 ±17	NS	90 ±111	116 ±139	NS	88 ±17	74 ±18	***
Diabetes mellitus	101 (26)	246 ±221	223 ±206	NS	93 ±17	90 ±17	NS	230 ±207	216 ±209	NS	93 ±13	90 ±18	NS	133 ±152	107 ±132	NS	82 ±18	73 ±18	***
Malignancy																			
Haematological	16 (4)	275 ±248	227 ±209	NS	90 ±19	91 ±17	NS	227 ±271	219 ±206	NS	90 ±12	91 ±17	NS	134 ±204	112 ±134	NS	77 ±21	75 ±19	NS
Non-haematological	42 (11)	217 ±224	230 ±209	NS	91 ±12	91 ±17	NS	250 ±226	215 ±206	NS	90 ±14	91 ±18	NS	121 ±148	112 ±136	NS	75 ±18	75 ±19	NS
Rheumatoid disease ^**C**^	46 (12)	295 ±273	220 ±199	NS	90 ±25	91 ±16	NS	239 ±240	217 ±204	NS	95 ±23	90 ±16	NS	155 ±154	109 ±135	NS	81 ±19	75 ±18	NS
Alcoholism	42 (11)	227 ±206	229 ±211	NS	94 ±16	91 ±17	NS	215 ±197	220 ±210	NS	95 ±20	90 ±17	NS	143 ±176	110 ±132	NS	82 ±18	75 ±19	*
**Severity of illness**																			
PITT score ≥ 3 ^**D**^	26 (7)	341 ±232	219 ±205	**	96 ±11	91 ±17	*	367 ±232	207 ±201	**	92 ±17	91 ±17	NS	141 ±94	110 ±134	*	81 ±17	75 ±20	NS
Severe sepsis ^**D**^	26 (7)	299 ±205	224 ±210	**	96 ±11	90 ±17	*	327 ±238	212 ±204	**	92 ±16	90 ±17	NS	130 ±129	113 ±137	NS	81 ±20	75 ±19	NS
Intensive care unit ^**D**^	59 (15)	277 ±214	218 ±206	**	97 ±9	89 ±18	*	280 ±232	206 ±199	**	93 ±15	90 ±18	NS	112 ±105	111 ±136	NS	79 ±15	75 ±19	NS
Any deep infection ^**E**^	330 (83)	237 ±208	188 ±86	**	92 ±17	86 ±19	*	230 ±209	161 ±191	**	91 ±18	87 ±14	*	116 ±134	101 ±152	*	77 ±18	67 ±21	**
Endocarditis ^**E**^	74 (19)	202 ±168	234 ±218	NS	93 ±13	91 ±17	NS	241 ±200	215 ±210	NS	91 ±14	91 ±18	NS	115 ±114	113 ±141	NS	77 ±16	75 ±19	NS
Prolonged fever ^**F**^	46 (12)	299 ±232	220 ±206	**	95 ±21	90 ±16	*	279 ±221	211 ±205	*	93 ±14	90 ±18	NS	181 ±196	105 ±125	*	79 ±21	75 ±18	NS
**Mortality**																			
At 14 days	31 (8)	358 ±291	218 ±199	**	94 ±13	91 ±17	NS	413 ±282	204 ±194	**	95 ±14	90 ±17	NS	---	---	---	---	---	---
At 28 days	50 (13)	312 ±264	217 ±199	**	94 ±14	90 ±17	NS	324 ±266	206 ±196	**	95 ±13	90 ±18	NS	---	---	---	---	---	---
At 90 days	72 (18)	282 ±247	216 ±198	*	93 ±14	90 ±18	NS	279 ±241	207 ±199	*	95 ±16	89 ±17	NS	171 ±221	109 ±128	*	89 ±15	74 ±18	NS

^**1**^ MMP-8 and TIMP-1 are presented and compared according to presence versus absence of the patient parameters in the left column.

* p < 0.05

** p < 0.01

*** p < 0.001 and NS = non-significant.

^**A**^ Classification according to McCabe and Jackson [38].

^**B**^ Chronically elevated creatinine (> 180 μmol/l).

^**C**^ Rheumatoid and/or connective tissue disease.

^**D**^At blood culture collection time-point.

^**E**^ During 90 days follow-up.

^**F**^ Duration ≥ 7 days.

Values are N (%) or mean (± standard deviation).

### Severity of illness and deep infection foci

A total of 7% of patients had severe sepsis and 15% needed ICU treatment at blood-culture collection time-point. High Pitt bacteremia score, severe sepsis and ICU treatment were associated to higher MMP-8 concentrations at day 3 and day 5 (p<0.01) and higher TIMP-1 at day 3 (p<0.05) whereas no connection to day TIMP-1 was observed. High Pitt bacteremia scores were associated to higher MMP-8 (p<0.05) on day 28 but TIMP-1 level was not affected by the Pitt bacteremia score (Table 1). Patients with any deep infection foci, as compared with patients without a deep infection foci, demonstrated higher MMP-8 and TIMP-1 levels in all sampling points whereas no differences in them were observed among patients who had endocarditis or not (Table 1).

### Antibiotic therapy

All patients were treated with an antibiotic effective *in vitro* against the *S*. *aureus* blood isolate starting from the day of the positive blood culture. Majority of the patients (75%) received an anti-staphylococcal penicillin (cloxacillin) whereas 18% got cefuroxime and 3% ceftriaxone. Vancomycin was used in 2% and it was the only antibiotic in 0.5% of the patients. Rifampicin or a fluoroquinolone was given to 58% or 59% of patients, respectively.

### Mortality and defervescence

**Fig 1:**
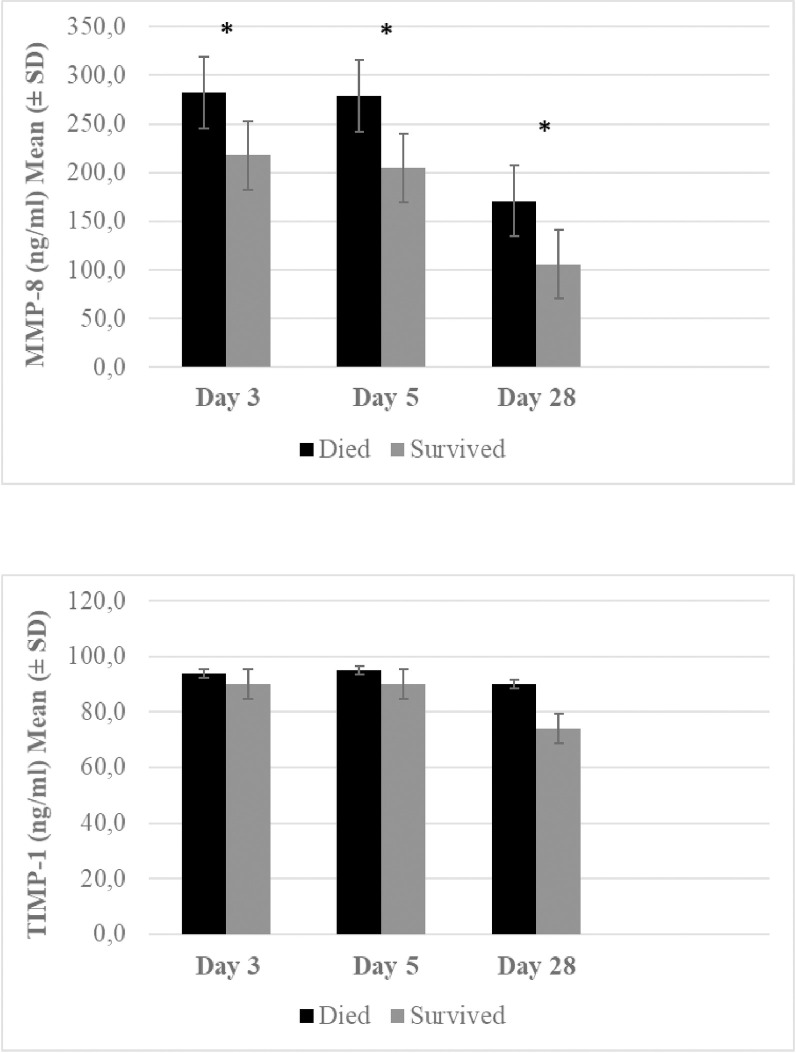
Comparison of matrix metalloproteinase 8 (MMP-8) and tissue inhibitor of metalloproteinase- 1 (TIMP-1) determined at day 3 (N = 395), day 5 (N = 378) and day 28 (N = 315) and categorized according to survival in methicillin-sensitive *Staphylococcus aureus* bacteremia. Data presented as mean (± standard deviation). * p < 0.05, ** p < 0.01, *** p < 0.001.

The primary end-point was mortality at 14 day and it occurred in 8% (31 patients) whereas the secondary end-point, mortality at 28 days and at 90 days occurred in 13% (50) and 18% (72) of patients, respectively (Table 1 and Fig 1). The MMP-8 levels at day 3 and 5 were higher among patients who deceased within 14 days (p<0.01), 28 days (p<0.01) and 90 days (p<0.05) as compared to surviving patients. However, no difference was seen in TIMP-1 levels between surviving and non-surviving patients. Altogether 22 patients (6%) died after the first month and among these patients the MMP-8 levels at day 28 were higher as compared to surviving patients (p<0.05) whereas no difference was seen in the TIMP-1 levels (Table 1).

The mean time to defervescence was 4.1 ± 5.4 days (mean ±SD) and 46 patients (12%) had fever longer than 7 days. Patients with prolonged fever, compared to patients with defervescence within 7 days, had higher MMP-8 levels at day 3 (p<0.01), day 5 (p<0.05) and day 28 (p<0.05) and higher TIMP-1 at day 3 (p<0.05) (Table 1). Furthermore, among background conditions chronic renal failure (N = 57) was the only factor associated significantly to lower MMP-8 levels (p<0.05) and higher TIMP-1 levels (p<0.01) on day 3. Hence, these patients were excluded from further statistical calculations and analyzed as a separate group. However, among patients with chronic renal failure, day 3, day 5 or day 28 MMP-8, TIMP-1 or MMP-8/TIMP-1 had no significant predictive effect on mortality when analyzed by receiver operating characteristic.

As a further analysis the patient population was stratified according to deceased or survived patients and patient characteristics and MMP-8 and MMP-8/TIMP-1 ratio compared (Table 2). Patients who deceased within 14 days, compared to survived ones, were more often aged > 60 years (77% vs. 49%, p<0.01), had less often healthy-nonfatal underlying conditions (35% vs. 75%, p<0.001) and more often ultimately-rapidly fatal underlying conditions (p<0.001) and were more often severely ill including need for ICU treatment (35% vs. 13%, p<0.01) and severe sepsis (23% vs. 5%, p<0.001) and presented more often endocarditis (35% vs. 15%, p<0.01). When comparing characteristics for deceased and survived patients during 28 and 90 days follow-up the results resembled closely those of 14 days follow-up (Table 2). Patients who deceased within 14 days had significantly higher median day 3 and 5 MMP-8 (p<0.01) and median day 3 MMP-8/TIMP-1 (p<0.05) and median day 5 MMP-8/TIMP-1 (p<0.05). Similar results were observed when comparing MMP-8 and MMP-8/TIMP-1 for patients who deceased and survived during 28 and 90 days follow-up. However, day 3 MMP-8/TIMP-1 did not differ between patients who deceased or survived 90 days follow-up (Table 2).

**Table 2:** Patient characteristics, illness severity and plasma matrix metalloproteinase-8 (MMP-8) and plasma matrix metalloproteinase-8 and tissue inhibitor of metalloproteinase-1 ratio (MMP-8 / TIMP-1) at day 3 and 5 from positive blood cultures in 395 patients with methicillin-sensitive *Staphylococcus aureus* bacteremia stratified according whether patients deceased or survived 90 days follow-up.

Patient characteristics	14 days follow-up				28 days follow-up				90 days follow-up			
	Deceased patients 31 (8)	Survived patients 364 (92)	OR (95% CI)	p-value	Deceased patients 50 (13)	Survived patients 345 (87)	OR (95% CI)	p- value	Deceased patients N = 72 (18)	Survived patients N = 323 (82)	OR (95% CI)	p-value
**Demographics**												
Male sex	20 (65)	228 (63)	1.09 (0.50–2.33)	NS	32 (64)	216 (63)	1.06 (0.57–1.97)	NS	44 (61)	204 (63)	0.92 (0.54–1.55)	NS
Age > 60 years	24 (77)	177 (49)	3.62 (1.52–8.62)	**	39 (78)	162 (47)	4.01 (1.99–8.08)	***	52 (72)	149 (46)	3.04 (1.73–5.32)	***
Healthcare-associated	16 (52)	193 (53)	0.95 (0.45–1.97)	NS	29 (58)	180 (52)	1.27 (0.69–2.31)	NS	47 (65)	162 (50)	1.87 (1.10–3.18)	*
**Underlying condition**												
Healthy-nonfatal ^**A**^	11 (35)	272 (75)	0.19 (0.09–0.40)	***	23 (46)	260 (75)	0.28 (0.15–0.51)	***	30 (42)	253 (78)	0.19 (0.12–0.34)	***
Ultimately-rapidly fatal ^**A**^	20 (65)	92 (25)	5.38 (2.48–11.6)	***	27 (54)	85 (25)	3.59 (1.96–6.59)	***	42 (58)	70 (22)	5.06 (2.95–8.67)	***
Coronary artery disease	10 (32)	93 (26)	1.39 (0.63–3.05)	NS	18 (36)	85 (25)	1.72 (0.92–3.22)	NS	25 (35)	78 (24)	1.67 (0.97–2.89)	NS
Diabetes mellitus	7 (23)	94 (26)	0.84 (0.35–2.01)	NS	12 (24)	89 (26)	0.91 (0.46–1.82)	NS	21 (29)	80 (25)	1.25 (0.71–2.21)	NS
Malignancy												
Haematological	3 (10)	13 (4)	2.89 (0.78–10.8)	NS	3 (6)	13 (4)	1.63 (0.45–5.93)	NS	6 (8)	10 (3)	2.85 (0.99–8.10)	*
Non-haematological	5 (16)	37 (10)	1.70 (0.62–4.69)	NS	8 (16)	34 (10)	1.74 (0.76–4.02)	NS	14 (19)	28 (9)	2.54 (1.26–5.12)	***
Rheumatoid disease ^**B**^	6 (19)	40 (11)	1.94 (0.75–5.03)	NS	11 (22)	35 (10)	2.50 (1.17–5.32)	NS	12 (17)	34 (11)	1.70 (0.83–3.47)	NS
Alcoholism	5 (16)	37 (10)	1.70 (0.62–4.69)	NS	9 (18)	33 (10)	2.08 (0.93–4.65)	NS	12 (17)	30 (9)	1.95 (0.95–4.03)	NS
**Severity of illness**												
PITT score ≥ 3 ^**C**^	9 (29)	17 (5)	8.72 (3.48–21.9)	***	10 (20)	16 (5)	5.41 (2.29–12.8)	***	12 (17)	14 (4)	4.57 (2.01–10.4)	***
Severe sepsis ^**C**^	7 (23)	19 (5)	5.30 (2.03–13.8)	***	10 (20)	16 (5)	5.14 (2.19–12.1)	***	11 (15)	15 (5)	3.70 (1.62–8.45)	**
Intensive care unit ^**C**^	11 (35)	48 (13)	3.79 (1.70–8.48)	**	15 (30)	44 (13)	3.11 (1.56–6.18)	**	19 (26)	40 (12)	2.64 (1.42–4.91)	**
Any deep infection ^**D**^	29 (94)	300 (82)	3.09 (0.72–13.3)	NS	48 (96)	281 (81)	5.47 (1.30–23.1)	*	68 (94)	261 (81)	4.04 (1.42–11.5)	**
Endocarditis ^**D**^	11 (35)	56 (15)	3.03 (1.37–6.65)	**	15 (30)	52 (15)	2.42 (1.23–4.73)	**	21 (29)	46 (14)	2.48 (1.37–4.50)	**
Prolonged fever ^**E**^	1 (3)	45 (12)	0.24 (0.03–1.78)	NS	5 (10)	41 (12)	0.82 (0.31–2.19)	NS	7 (10)	39 (12)	0.78 (0.34–1.83)	NS
Day 3												
MMP-8 ^**1**^	210 (148–604)	163 (76.3–277)	---	**	204 (142–530)	163 (74–278)	---	**	199 (121–347)	161 (74.4–278)	---	*
MMP-8 / TIMP-1 ^**1**^	1.08 (0.63–2.93)	0.76 (0.36–1.31)	---	*	0.93 (0.61–2.28)	0.76 (0.35–1.32)	---	*	0.89 (0.59–1.73)	0.76 (0.35–1.33)	---	NS
Day 5												
MMP-8 ^**1**^	327 (149–736)	138 (77.6–256)	---	**	240 (100–522)	138 (78–257)	---	**	200 (94.1–408)	138 (74.4–256)	---	*
MMP-8 / TIMP-1 ^**1**^	1.61 (0.71–3.12)	0.71 (0.36–1.21)	---	**	1.15 (0.47–2.35)	0.71 (0.36–1.22)	---	**	1.02 (0.45–1.75)	0.71 (0.36–1.19)	---	*

* p < 0.05

** p < 0.01

*** p < 0.001 and NS = non-significant.

^**1**^ Median and IQR.

^**A**^ Classification according to McCabe and Jackson [38].

^**B**^ Rheumatoid and/or connective tissue disease.

^**C**^At blood culture collection time-point.

^**D**^ During 90 days follow-up.

^**E**^ Duration ≥ 7 days.

Values are N (%) or median (+ IQR).

When applying the generalized linear model repeated measure analyze for comparison of MMP-8, TIMP-1 and MMP-8/TIMP-1 throughout the 90 days follow-up period for patients who deceased versus those that survived, only the MMP-8 levels were significantly different (p-value<0.01) whereas the TIMP-1 or MMP-8/TIMP-1 levels did not differ significantly.

### Cut-off values for MMP-8 and MMP-8/TIMP-1 in predicting mortality

**Fig 2:**
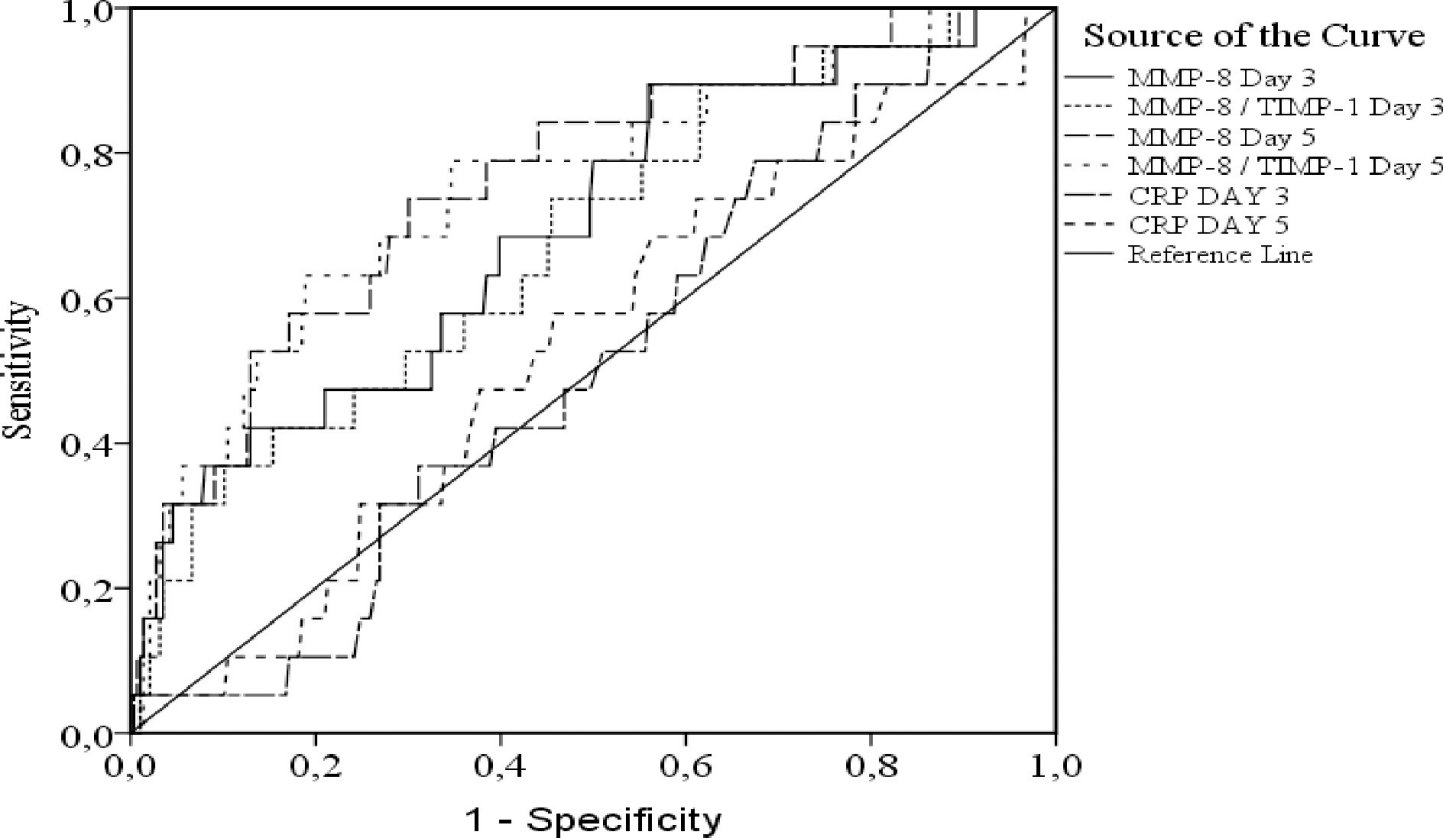
Receiver operating characteristic (ROC) curves for predicting 14 days mortality in methicillin-sensitive *Staphylococcus aureus* bacteremia. Matrix metalloproteinase 8 (MMP-8), matrix metalloproteinase 8 and tissue inhibitor of metalloprotein- ase-1 ratio (MMP-8 / TIMP-1) and C-reactive protein (CRP) were measured at day 3 (N = 395) and 5 (N = 378) past positive blood cultures. Patients with chronic renal failure were excluded (N = 57) due to low initial MMP-8 concentrations. For day 3 the MMP-8 area under the curve (AUC) was 0.70 (95% Cl 0.57–0.82) (p<0.01) with cut-off value 203 ng/ml and sensitivity 68% and specificity 60%. For day 3 MMP-8 / TIMP-1 ratio the AUC was 0.68 (95% Cl 0.55–0.80) (p<0.01) with cut of value 0.87 and sensitivity 74% and specificity 56%. For day 5 MMP-8 the AUC was 0.76 (95% Cl 0.65–0.87) (p<0.001) with cut-off value of 239 ng/ml with sensitivity 74% and specificity 70%. For day 5 MMP-8 / TIMP-1 ratio the AUC was 0.75 (95% Cl 0.63–0.87) (p<0.001) with cut-off valué 1.02 and sensitivity 79% and specificity 66%. For day 3 and day 5 CRP the AUCs were 0.51 (95% Cl 0.40–0.63) (p = 0.85) and 0.53 (95% Cl 0.40–0.66) (both p-values non-significant).

To study the potential pathogenetic role of MMP activation in SAB we analyzed the predictive value of MMP-8 and MMP/TIMP-1 ratio at day 3 and 5 on mortality within 14, 28 or 90 days and compared it to C-reactive protein. MMP-8 and MMP-8/TIMP-1 ratio at day 5 were the strongest predictors of mortality within 14 days (Fig 2). The ROC analysis for day 5 MMP-8 for predicting death within 14 days was 0.76 (95% CI 0.65–0.87) (p<0.001) with cut-off value of 239 ng/ml with sensitivity of 74% and specificity of 70% whereas the ROC analysis for day 5 MMP-8/TIMP-1 ratio for predicting death within 14 days was 0.75 (95% CI 0.63–0.87) (p<0.001) with cut-off value 1.02 and sensitivity 79% and specificity 66% (Fig 2). Day 3 and day 5 MMP-8 levels predicted mortality within 28 days and day 5 MMP-8 level predicted mortality within 90 days, however, the AUCs and sensitivity and specificity were lower than for predicting 14 days mortality (S1 and S2 Figs). CRP was not predictive for mortality (Fig 2 and S1 and S2 Figs).

### Cox proportional regression model analysis

Parameters predicting 14 days mortality were evaluated together with day 5 MMP-8 and MMP-8/TIMP-1 cut-off values by applying Cox proportional regression analysis (Table 3). Healthy-nonfatal underlying disease was linked to lower 14 day mortality (HR 0.25, p<0.01), but ICU treatment (HR 3.24, p<0.05) and day 5 MMP-8/TIMP-1 molar ratio >1.02 were associated to higher mortality within 14 days (HR 4.71, p<0.01). Additional analyzes for parameters predicting day 28 and day 90 mortality were performed (S1 Table). Age > 60 years (HR 2.72, p<0.05), ICU treatment (HR 2.91, p<0.01), endocarditis (HR 2.61, p<0.05) and day 5 MMP-8/TIMP-1 molar ratio >1.02 (HR 2.34, p<0.05) predicted mortality at 28 days whereas healthy-nonfatal underlying disease (HR 0.43, p<0.05) and rifampicin adjunctive therapy (HR 0.26, p<0.05) improved prognosis. ICU treatment (HR 2.12, p<0.05) and endocarditis (HR 2.36, p<0.05) were connected to higher 90 days mortality whereas healthy-nonfatal underlying disease (HR 0.23, p<0.010) and rifampicin adjunctive therapy (HR 0.38, p<0.01) were associated to improved prognosis. Day 5 MMP-8 or MMP-8/TIMP-1 molar ratio did not impact 90 day mortality (S1 Table).

**Table 3:** Prognostic factors for 14-days mortality in patients with methicillin-sensitive *Staphylococcus aureus* bacteremia (N = 338).

Patient characteristics	14-days mortality		Univariate analysis		Cox regression	
	Died n = 22 (6)	Survived n = 316 (94)	OR (95% CI)	p-value	HR (95% CI)	p-value
Male sex	16 (73)	192 (61)	1.72 (0.66–4.52)	NS	---	---
Age > 60 years	16 (73)	149 (47)	2.99 (1.14–7.84)	*	---	---
Healthy—nonfatal ^**A**^	11 (50)	263 (83)	0.20 (0.08–0.49)	***	0.25 (0.10–0.63)	**
Intensive care unit ^**B**^	11 (50)	44 (14)	6.78 (2.72–16.9)	***	3.24 (1.29–8.11)	*
MMP-8 cut-off 203 ^**C**^	14 (64)	125 (39)	2.67 (1.09–6.56)	*	---	---
MMP-8 cut-off 239 ^**D**^	15 (68)	88 (28)	6.99 (2.46–19.8)	***	---	---
MMP-8 / TIMP-1 cut-off 0.87 ^**C**^	16 (73)	148 (47)	3.03 (1.15–7.94)	*	---	---
MMP-8 / TIMP-1 cut-off 1.02 ^**D**^	16 (73)	101 (32)	7.60 (2.48–23.3)	***	4.71 (1.67–13.3)	**
Endocarditis	9 (41)	51 (16)	3.59 (1.46–8.86)	**	---	---

* p < 0.05

** p < 0.01

*** p < 0.001 and NS = non-significant.

^**A**^ McCabe’s classification [38].

^**B**^ At blood culture collection time-point.

^**C & D**^ At day 3 and 5 past blood culture collection.

^**E**^ Adjunctive therapy.

Patients with chronic renal failure were excluded (N = 57) due to low initial MMP-8 concentrations. Values are number of patients (%), odds or hazards ratios (OR or HR) with 95% confidence intervals (95% CI).

### Additional analyzes for TIMP-1

No association of TIMP-1 to mortality was observed in ROC-analyzes (Fig 2 and S1 and S2 Figs) with AUCs varying from 0.25–0.75 and 95% CI intervals varying between 0.031 and 0.97 and p-value non-significant for each measurement. To further evaluate the prognostic role of TIMP-1 we included only patients with ICU at blood culture collection or within 3 days, however, by this subgrouping the TIMP-1 had no connection to 14, 28 or 90 days mortality when analyzed by univariate analysis (p-value non-significant for each measurement).

## Discussion

The main observation of the present study was that plasma MMP-8 and MMP-8/TIMP-1 molar ratio determined at an early stage (day 3 and 5 after positive blood-cultures) were significantly higher in SAB patients with a more severe disease i.e. severe sepsis, ICU treatment need and patients with a deep infection focus as compared to patients without these complications. Interestingly, higher MMP-8 and TIMP-1 levels were associated to presence of a deep infection focus but not to endocarditis. Higher MMP-8 levels on day 3 and especially on day 5 were not only associated to a more severe disease but were also predictive for mortality within 14 and 28 days. When all prognostic parameters were controlled for in Cox regression analysis high day 5 MMP-8/TIMP-1 ratio predicted mortality within 14 or 28 days but not at a later stage as analyzed by day 90 mortality. No association to mortality was seen for the commonly used inflammatory marker CRP. This data suggests that matrix metalloproteinase activation may be one link in the pathological processes in complicated SAB.

Comparison of results from the present study to previous reports on the prognostic impact of MMPs and TIMPs in infections or critically ill patients is challenging due to variations in patient profiles, categorization according to illness, exclusion criteria, time-point of MMP or TIMP measurement and follow-up times. Furthermore, the present study is the only one that has included solely bacteremia patients due to one causative agent in contrast to sepsis or other critical illnesses in previous reports.

First, we included only MS-SAB patients and MMP-8 and TIMP-1 concentrations were measured in relation to bacteremia onset regardless of severity of illness, PITT bacteremia score or ICU admission. In the present study only 7% had severe sepsis and 15% needed ICU treatment. In contrast, many previous studies have included only septic and/or critically ill patients [13,14,25–34] or only patients with acute respiratory failure [15] and MMPs and TIMPs have commonly been determined at sepsis diagnosis or ICU admission [13–15,25–31,33–34]. Furthermore, we determined MMPs and TIMPs at days 3, 5 and 28 after positive blood culture i.e. several measurement points whereas most previous reports have determined MMPs and TIMPs at sepsis onset and/or ICU admission only. To the best of our knowledge only one report has determined MMP-2, -8 and -9 in severe sepsis on days 1, 4, 6, 8 and 10 and from survivors at 3 and 6 months from hospital admission [32]. Thus, it is plausible to assume that MMPs and TIMPs in previous studies have been determined at a time-point when the patients have been more severely ill. This is probably the major explanation for the much higher mortality rates reported in previous studies: 13–34% overall sepsis or ICU mortality [25,29] and 12–33% 30-days mortality or 27% 90-day mortality in septic and/or critically ill patients [13–15,25–34] as compared to 13% and 18% mortality rates at 28 and 90 days in the present study.

Second, we observed that chronic renal failure was associated to significantly lower day 3 MMP-8 levels and higher day 3 TIMP-1 levels. Moreover, among patients with chronic renal failure MMP-8, TIMP-1 and MMP-8/TIMP-1 levels had no prognostic impact in SAB. Thus, patients with chronic renal failure were excluded from the main analyzes. Previous reports on MMPs and/or TIMPs have analyzed patients as one group [14,25,28,30,33] or in specific subgroups including e.g. solely acute respiratory distress syndrome [15] or multi-organ failure patients [32]. Moreover, many reports on MMPs and TIMPs in sepsis or critical illness have excluded immunocompromised patients or patients with malignancies or liver disease [13,15,26,27,29]. However, we are not aware of reports on MMPs or TIMPs levels in renal failure or of reports excluding patients with renal failure.

Third, the present study included only bacteremia due to methicillin sensitive *S*. *aureus* and each patient received effective antimicrobial therapy from the day when the positive blood culture was drawn. Among previous reports on MMPs and TIMPs in septic or critically ill patients only three studies have reported that 13% - 50% of septic patients have had positive blood cultures with two thirds having streptococcal and one third of miscellaneous bacterial etiology [27] or positive blood cultures for *Bacteroides fragilis* or *Candida albicans* [31] whereas one report did not mention the pathogens [26]. One study reported that 7% of patients had blood as a primary focus of infection [32]. Delayed effective antimicrobial therapy is a risk factors for poor prognosis [45]. MRSA is associated with poor prognosis and delay in effective therapy [9,46]. Vancomycin treatment increases the risk for persistent SAB compared to treatment with anti-staphylococcal penicillin [47]. Furthermore, all patients received formal ISC which is known to improve clinical management and prognosis of SAB patients [6,7]. All these pitfalls/sources of bias were controlled for in our study setup.

Fourth, the present study included only MS-SAB patients. Thus, the study cannot evaluate how MMP-8, TIMP-1 and MMP-8/TIMP-1 would perform in MRSA bacteremia. However, there were only 6 MRSA bacteremia patients and thus the excluded MRSA bacteremia patients represented only 1.3% (6/430) of the whole patient cohort. Hence, it is plausible to assume that this exclusion had not significantly affected the results.

The present study observed significantly higher early MMP-8 (day 3 and 5) and TIMP-1 (day 3) levels among patients with severe sepsis, high Pitt bacteremia scores and ICU treatment compared to patients without severe illness or need for ICU. However, this trend declined during follow-up and at day 28 only high Pitt bacteremia scores were connected to elevated MMP-8 levels. These observations are in line with many previous studies reporting elevated levels of circulating MMPs and TIMPs in septic and/or critically ill patients as compared to controls [13–15, 25–34]. Six reports including 20 to 563 patients with severe sepsis or septic shock determined various MMPs and TIMPs at ICU admission [25], on day 1 of illness [28] or on days 1, 4, 6, 8, 10 and at 3 and 6 months from hospital admission [32] or at time-point of diagnosis [29,33]. Higher MMP-2 [32], MMP-8 [25,32], MMP-9 [25], MMP-10 [29], TIMP-1 [25,28,29] and MMP-10/TIMP-1 [29] were seen in severe sepsis compared to controls. Previous reports have presented mean or median MMP-8 levels of 49–71 ng/mL and 93 ng/mL, TIMP-1 mean or median levels of 310–413 ng/mL and 226–1687 ng/mL and median MMP-8/TIMP-1 molar ratios of 0.12 in patients with sepsis or critical illness at hospital or ICU admission or at sepsis onset whereas in the present study the mean day 3, 5 and 28 MMP-8 levels were higher and TIMP-1 levels were lower than earlier reported in sepsis or critical illness 13–15,23,25,26,29].

Due to meticulous clinical and radiological investigations and ISC for each patient a total of 83% had a deep infection focus and 19% endocarditis diagnosed. We observed a strong connection of deep infection foci to higher MMP-8 and TIMP-1 levels throughout the 90 days observation period. However, this trend was not seen for endocarditis. Previous reports on MMPs and TIMPs in septic or critically ill patients have presented high occurrence of infection foci including 16–57% pneumonia, 4% endocarditis and 27–36% abdominal focus, however, the explicit connection of MMPs and TIMPs or MMP/TIMP molar ratio have not been evaluated in these previous reports [15,26,32].

In the present study, day 3, 5 and 28 MMP-8 levels were significantly higher in patients who deceased within 14, 28 or 90 days as compared to survivors. No corresponding trend at was seen for TIMP-1. Previous reports have observed higher MMP-8 on [25] and TIMP-1 [13,23,25,29] at sepsis onset or ICU admission in non-surviving sepsis patients compared to surviving ones. Furthermore, two reports observed that critically ill non-surviving patients at ICU admission or in need of mechanical ventilation, as compared to surviving ones, presented higher MMP-8 and TIMP-1 levels [14,15] but indifferent MMP-8/TIMP-1 molar ratios [15]. Hence, the observations in the present report of higher MMP-8 among non-surviving patients are in line with previous observations [25] whereas the lack of association of TIMP-1 to mortality is in contrast with many previous observations [13,23,25,29].

By ROC analyzes significantly higher day 3 MMP-8 levels were observed in patients who deceased early (within 14 or 28 days) and in day 5 MMP-8 levels of deceased patients regardless of time of death. Day 3 MMP-8/TIMP-1 molar ratio was associated to mortality at an early phase (within 14 days) whereas day 5 MMP-8/TIMP-1 predicted mortality at 14 and 28 days. Few previous reports have performed ROC analyzes to determine the capability of MMP-8, TIMP-1 and MMP-8/TIMP-1 to discriminate surviving and non-surviving septic or critically ill patients. To the best of our knowledge, three reports determined MMP-8 and TIMP-1 at hospital or ICU admission in septic or critically ill patients and presented TIMP-1 sensitivity 60–73% and specificity 45–76% with AUC 0.62–69 and cut-off values 331–531 ng/mL and MMP-8 sensitivity 49% and specificity 60% with AUC 0.55 and cut-off value 127 ng/mL in predicting 30–90 days mortality or overall sepsis mortality [13,15,29]. These results differ partly from the observations of the present study. First, we observed no connection of TIMP-1 to mortality. Second, the ROC analysis results for admission MMP-8 in predicting 90 days mortality in critically ill acute respiratory failure patients resembled the results in the present study where day 5 MMP-8 predicted death within 90 days with an AUC of 0.61, cut-off 149 ng/mL, sensitivity and specificity of 65% and 54% [15].

The observations in univariate and Cox proportional analyzes in the present study of age > 60 years, healthy-nonfatal underlying conditions, ICU treatment, endocarditis and adjunctive rifampicin therapy as parameters with strong prognostic impact have been reported earlier [2,3,4,48]. In univariate analysis, a strong connection of day 3–5 MMP-8 and MMP-8/TIMP-1 to 14 and 28 days mortality was observed. However, only day 5 MMP-8 and MMP-8/TIMP-1 -ratio were associated to 90 days mortality. When accounting for all prognostic parameters in Cox proportional regression analysis, day 5 MMP-8/TIMP-1 molar ratio was a strong predictor of 14- and 28-days but not 90-days mortality. However, day 3–5 MMP-8 did not predict mortality in Cox proportional regression analyzes and TIMP-1 was not included in this analysis as no connection of TIMP-1 to mortality was observed in the original univariate analysis. Previous reports have presented admission TIMP-1 as an independent negative prognostic marker for 30–90 days or overall mortality in critically ill, severe sepsis or acute respiratory failure patients [14,15,23,27,29]. These observations are in sharp contrast with the results of the present study but in line with one study that observed no significant predictive value of admission TIMP-1 in septic patients on 30 day mortality [13].

The results of the present study demonstrate a connection of MMP-8 and MMP-8/TIMP-1 and disease severity and mortality in SAB. This suggest that matrix metalloproteinase activation may be one link in the pathological processes linked to disease severity and mortality in SAB. Previous results in septic or critically ill patients suggest that MMP activation may play a role in pathologic processes in bacteremia and critical illness. However, the sensitivity and specificity of the MMP measurements in predicting mortality have been fairly low and suggest that they might not be useful as clinical markers. The possibility to affect MMP activation pharmacologically make these observations interesting and encourage further studies on the role of MMP activation in bacteremia and critical illness [20].

There are limitations and weaknesses of the present study that have to be taken into account when interpreting the results. The patient cohort of the present study was prospectively collected in 1999–2002, however, the MMP-8 and TIMP-1 laboratory analyzes were not performed until year 2015–2016. Clinical practice of SAB develops continuously, however, there are fundamental elements of SAB treatment that have remain unchanged e.g. the importance of non-delayed onset of proper antibiotic treatment and eradication of deep infection foci. The authors view that bedside formal infectious diseases specialist consultation in the present study has guaranteed recording of relevant clinical patient information and hence enabled high standard clinical management of SAB. Laboratory quality testing have ensured that MMP and TIMP remained stable at –80°C and hence the standard of the laboratory samples are guaranteed despite longtime storage. Hence, the authors view that the patient data of the present study is not outdated and the laboratory samples have been correctly stored and analyzed and the results are reliable.

In conclusion, the present study is the first to demonstrate a connection of plasma MMP-8 and MMP-8/TIMP-1 to disease severity, presence of a deep infection focus and mortality in MS-SAB patients. However, comparison of the results to previous studies on MMPs and TIMPs in sepsis or critical illness is challenging due to differences in patient populations. Future studies need to evaluate the pathogenetic role of MMP-8 and MMP-8/TIMP-1 in bacteremia which might open possibilities for pharmacological therapies to control their activation in bacteremia.

## Supporting information

S1 Fig Receiver operating characteristic (ROC) curves for predicting 28 days mortality in methicillin-sensitive *Staphylococcus aureus* bacteremia (N = 339). Matrix metalloproteinase 8 (MMP-8), matrix metalloproteinase 8 and tissue inhibitor of metal- loproteinase-1 ratio (MMP-8 / TIMP-1) and C-reactive protein (CRP) were measured at day 3 (N = 395) and 5 (N = 378) past positive blood cultures. Patients with chronic renal failure were excluded (N = 57) due to low initial MMP-8 concentrations. For day 3 the MMP-8 area under the curve (AUC) was 0.61 (95% CI 0.51–0.72) (p<0.05) with cut-off value 167 ng/ml and sensitivity 66% and specificity 50%. For day 3 MMP-8 / TIMP-1 ratio the AUC was AUC 0.60 (95% CI 0.49–0.70) (p-value NS). For day 5 MMP-8 the AUC was 0.65 (95% CI 0.54–0.76) (p<0.01) with cut-off value 195 ng/ml and sensitivity 62% and specificity 61%. For day 5 MMP-8 / TIMP-1 ratio the AUC was 0.63 (95% CI 0.51–0.74) (p<0.05) with cut-off value 0.85 and sensitivity 62% and specificity 57%. For day 3 and day 5 CRP the AUCs were 0.55 (95% CI 0.44–0.66) and 0.53 (95% CI 0.42–0.64) (both p-values NS). NS = non-significant.
(TIF)

S2 Fig Receiver operating characteristic (ROC) curves for predicting 90 days mortality in methicillin-sensitive *Staphylococcus aureus* bacteremia (N = 339). Matrix metalloproteinase 8 (MMP-8), matrix metalloproteinase 8 and tissue inhibitor of metal- loproteinase-1 ratio (MMP-8 / TIMP-1) and C-reactive protein (CRP) were measured at day 3 (N = 395) and 5 (N = 378) past positive blood cultures. Patients with chronic renal failure were excluded (N = 57) due to low initial MMP-8 concentrations. For day 3 the MMP-8 area under the curve (AUC) was 0.58 (95% Cl 0.49–0.67) (p-value NS). For day 3 MMP-8 / TIMP-1 ratio the AUC was 0.57 (95% Cl 0.48–0.66) (p-value NS). For day 5 MMP-8 the AUC was 0.61 (95% Cl 0.52–0.69) (p<0.05) with cut-off value 149 ng/ml and sensitivity 65% and specificity 54%. For day 5 MMP-8 / TIMP-1 ratio the AUC was 0.59 (95% Cl 0.49–0.68) (p-value NS). For day 3 and day 5 CRP the AUCs were 0.47 (95% Cl 0.38–0.56) and 0.48 (95% Cl 0.39–0.57) (both p-values NS). NS = non-significant.
(TIF)

S1 Table Prognostic factors for 28- and 90-days mortality in patients with methicillin-sensitive *Staphylococcus aureus* bacteremia (N = 338). Patients with chronic renal failure were excluded (N = 57) due to low initial MMP-8 concentrations. Values are number of patients (%), odds or hazards ratios (OR or HR) with 95% confidence intervals (95% CI).
(DOCX)

S1 Data (SAV)
