# Supplementary figures and images for: Matrix metalloproteinase MMP-8, TIMP-1 and MMP-8/TIMP-1 ratio in plasma in methicillin-sensitive Staphylococcus aureus bacteremia
Source: PLoS One. 2021 May 27;16(5):e0252046. doi: 10.1371/journal.pone.0252046 (PMC8158883; doi:10.1371/journal.pone.0252046)

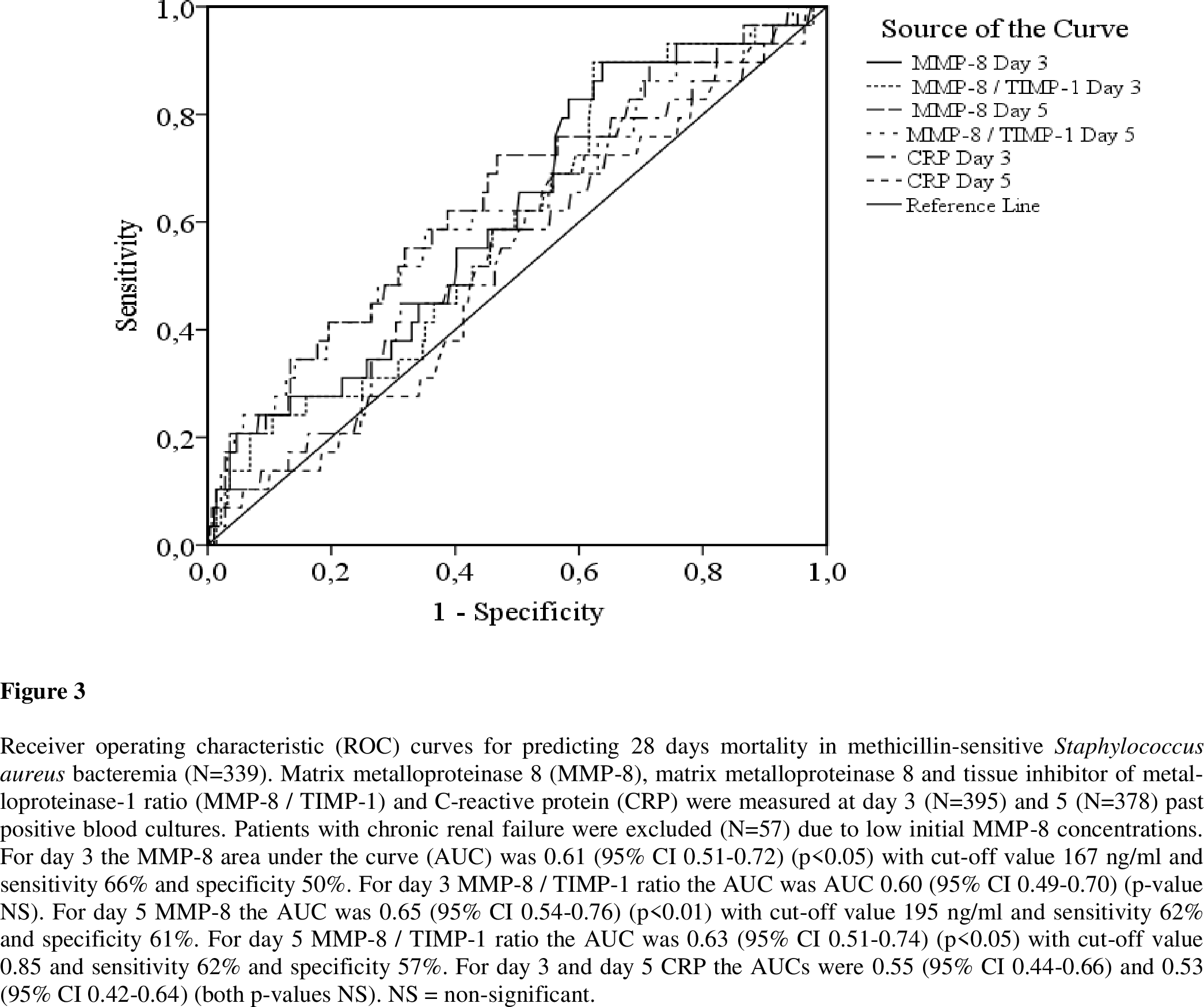

Supplement: S1 Fig — Matrix metalloproteinase 8 (MMP-8), matrix metalloproteinase 8 and tissue inhibitor of metal- loproteinase-1 ratio (MMP-8 / TIMP-1) and C-reactive protein (CRP) were measured at day 3 (N = 395) and 5 (N = 378) past positive blood cultures. Patients with chronic renal failure were excluded (N = 57) due to low initial MMP-8 concentrations. For day 3 the MMP-8 area under the curve (AUC) was 0.61 (95% CI 0.51–0.72) (p<0.05) with cut-off value 167 ng/ml and sensitivity 66% and specificity 50%. For day 3 MMP-8 / TIMP-1 ratio the AUC was AUC 0.60 (95% CI 0.49–0.70) (p-value NS). For day 5 MMP-8 the AUC was 0.65 (95% CI 0.54–0.76) (p<0.01) with cut-off value 195 ng/ml and sensitivity 62% and specificity 61%. For day 5 MMP-8 / TIMP-1 ratio the AUC was 0.63 (95% CI 0.51–0.74) (p<0.05) with cut-off value 0.85 and sensitivity 62% and specificity 57%. For day 3 and day 5 CRP the AUCs were 0.55 (95% CI 0.44–0.66) and 0.53 (95% CI 0.42–0.64) (both p-values NS). NS = non-significant. (TIF) [file pone.0252046.s001.tif]

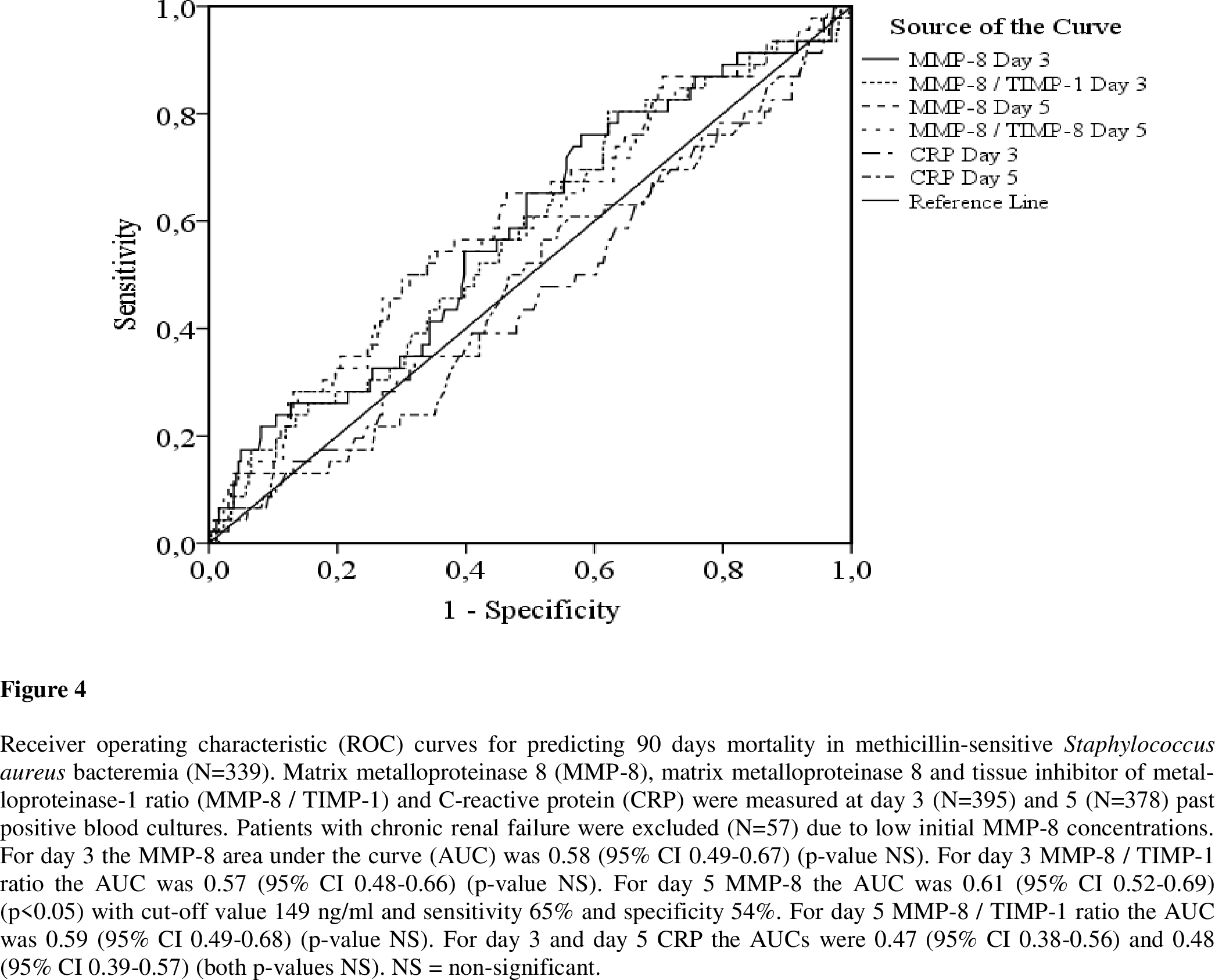

Supplement: S2 Fig — Matrix metalloproteinase 8 (MMP-8), matrix metalloproteinase 8 and tissue inhibitor of metal- loproteinase-1 ratio (MMP-8 / TIMP-1) and C-reactive protein (CRP) were measured at day 3 (N = 395) and 5 (N = 378) past positive blood cultures. Patients with chronic renal failure were excluded (N = 57) due to low initial MMP-8 concentrations. For day 3 the MMP-8 area under the curve (AUC) was 0.58 (95% Cl 0.49–0.67) (p-value NS). For day 3 MMP-8 / TIMP-1 ratio the AUC was 0.57 (95% Cl 0.48–0.66) (p-value NS). For day 5 MMP-8 the AUC was 0.61 (95% Cl 0.52–0.69) (p<0.05) with cut-off value 149 ng/ml and sensitivity 65% and specificity 54%. For day 5 MMP-8 / TIMP-1 ratio the AUC was 0.59 (95% Cl 0.49–0.68) (p-value NS). For day 3 and day 5 CRP the AUCs were 0.47 (95% Cl 0.38–0.56) and 0.48 (95% Cl 0.39–0.57) (both p-values NS). NS = non-significant. (TIF) [file pone.0252046.s002.tif]
