# Supplementary material for: Matrix metalloproteinase MMP-8, TIMP-1 and MMP-8/TIMP-1 ratio in plasma in methicillin-sensitive Staphylococcus aureus bacteremia
Source: PLoS One. 2021 May 27;16(5):e0252046. doi: 10.1371/journal.pone.0252046 (PMC8158883; doi:10.1371/journal.pone.0252046)
Supplement: S1 Table — Patients with chronic renal failure were excluded (N = 57) due to low initial MMP-8 concentrations. Values are number of patients (%), odds or hazards ratios (OR or HR) with 95% confidence intervals (95% CI). (DOCX) [file pone.0252046.s003.docx]

**Table 4** Prognostic factors for 28- and 90-days mortality in patients with methicillin-sensitive *Staphylococcus aureus* bacteremia (N=338). Patients with chronic renal failure were excluded (N = 57) due to low initial MMP-8 concentrations. Values are number of patients (%), odds or hazards ratios (OR or HR) with 95% confidence intervals (95% CI).

| **Patient**  **characteristics** | **28-days mortality** | | **Univariate analysis** | | **Cox regression** | |
| --- | --- | --- | --- | --- | --- | --- |
|  | Died  n=37 (11) | Survived  n=301 (89) | OR  (95% CI) | p-  value | HR  (95% CI) | p-  value |
| Male sex  Age > 60 years  Healthy - nonfatal ^A^  Intensive care unit ^C^  MMP-8 cut-off 203 ^C^  MMP-8 cut-off 239 ^D^  MMP-8 / TIMP-1 cut-off 0.87 ^C^  MMP-8 / TIMP-1 cut-off 1.02 ^D^  Endocarditis  Rifampicin | 25 (68)  28 (76)  23 (62)  14 (38)  21 (57)  16 (43)  23 (62)  18 (49)  12 (32)  9 (24) | 183 (61)  137 (45)  251 (83)  41 (14)  118 (39)  87 (29)  141 (47)  99 (33)  48 (16)  151 (50) | 1.34 (0.65-2.78)  3.72 (1.70-8.16)  0.33 (0.16-0.68)  4.23 (1.99-8.97)  2.04 (1.02-4.06)  2.58 (1.20-5.51)  1.86 (0.92-3.76)  2.79 (1.29-6.02)  2.53 (1.19-5.38)  0.32 (0.15-0.70) | NS  **  **  ***  *  **  *  **  *  ** | ---  2.72 (1.11-6.67)  0.43 (0.19-0.94)  2.91 (1.32-6.40)  ---  ---  ---  2.34 (1.09-5.03)  2.61 (1.14-5.94)  0.26 (0.11-0.62) | ---  *  *  **  ---  ---  ---  *  *  * |

| **Patient**  **characteristics** | **90-days mortality** | | **Univariate analysis** | | **Cox regression** | |
| --- | --- | --- | --- | --- | --- | --- |
|  | Died  n=54 (16) | Survived  n=284 (84) | OR  (95% CI) | p-  value | HR  (95% CI) | p-  value |
| Male sex  Age > 60 years  Healthy - nonfatal ^A^  Intensive care unit ^C^  MMP-8 cut-off 203 ^C^  MMP-8 cut-off 239 ^D^  MMP-8 / TIMP-1 cut-off 0.87 ^C^  MMP-8 / TIMP-1 cut-off 1.02 ^D^  Endocarditis  Rifampicin | 34 (63)  38 (70)  29 (54)  18 (33)  28 (52)  23 (43)  31 (57)  26 (48)  17 (31)  18 (33) | 174 (61)  127 (45)  245 (86)  38 (13)  111 (39)  89 (31)  133 (47)  91 (32)  43 (15)  142 (50) | 1.08 (0.59-1.96)  2.94 (1.57-5.51)  0.19 (0.09-0.35)  3.24 (1.67-6.27)  1.68 (0.94-3.01)  2.13 (1.14-3.97)  1.53 (0.85-2.75)  2.26 (1.21-4.21)  2.58 (1.33-4.98)  0.50 (0.27-0.92) | NS  **  ***  ***  NS  *  NS  **  **  * | ---  ---  0.23 (0.13-0.41)  2.12 (1.11-4.07)  ---  ---  ---  ---  2.36 (1.23-4.52)  0.38 (0.21-0.71) | ---  ---  ***  *  ---  ---  ---  ---  *  ** |

* p < 0.05, ** p < 0.01, *** p < 0.001 and NS = non-significant.

**^A^** McCabe’s classification [38]

**^B^** At blood culture collection time-point

**^C & D^** At day 3 and 5 past blood culture collection

**^E^** Adjunctive therapy
